# Estimation of Mercury Emission from Incineration of Extracted Teeth with Dental Amalgam Fillings in South Korea

**DOI:** 10.3390/ijerph15071325

**Published:** 2018-06-25

**Authors:** Hae-Jin Kim, Ji-Hye Park, Joon Sakong

**Affiliations:** 1 Department of Public Health, Graduate School of Yeungnam University, 170 Hyunchung-ro, Nam-gu, Daegu 42415, Korea; gowls0412@hanmail.net; 2 Department of Preventive Medicine & Public Health, College of Medicine, Yeungnam University, 170 Hyunchung-ro, Nam-gu, Daegu 42415, Korea; jhpark23@ynu.ac.kr

**Keywords:** dental amalgam, dental clinics, mercury, tooth extraction

## Abstract

This study is aimed to estimate the levels of mercury emission related to amalgam fillings at dental institutions in order to provide basic data for establishing a management protocol for extracted mercury-containing amalgam fillings. We conducted a cross-sectional study at a dental clinic of a general hospital (City of Daegu), a dental hospital (City of Daegu), and five private dental clinics (one in the South and four in the North Gyeongsang Province). The extracted anterior and posterior teeth (*N* = 1208) were separated, and the amalgam filling rate of the posterior teeth was assessed. After cutting out the amalgam from filled posterior teeth, the weight of the amalgam was measured, and the estimated mercury emission was calculated based on the equation, where annual number of extracted posterior teeth × amalgam filling rate (%) × mean amalgam weight (g) × 0.5 (the proportion of mercury in amalgam). We found that 48.86 kg (95% confidence interval [CI] 41.53–58.63 kg) of mercury had been incinerated along with the extracted teeth. After applying the dental institution weights, the estimated amount of mercury was 42.53 kg (95% CI 34.11–52.17 kg). The amount of mercury incinerated with extracted posterior teeth at Korean dental institutions is therefore about 42.53–48.86 kg/year.

## 1. Introduction

The amount of mercury used in dental amalgam in dental offices worldwide was around 272 t in 2000 [1]; annually, about 260–300 t of mercury from dental amalgam is emitted into the environment. This emitted mercury is released into the atmosphere (50–70 t), surface water (35–45 t), ground water (20–25 t), soil (75–100 t), and as waste (40–50 t) [2]. Approximately 40–50 t of mercury, which accounts for only 1/6 of the total mercury used in dental amalgam, is recycled worldwide annually [2].

Large amounts of emitted mercury can easily spread between countries and even continents; therefore, an international response to the emission and spread of mercury is essential. The need for such an international response had been introduced during the 2009 United Nations Environment Programme (UNEP) meeting; thereafter, the Minamata convention was established in 2013 to regulate the entire distribution process of mercury, including synthesis, storage, emission, and disposal. As of September 2016, 128 countries have agreed to comply with the Minamata convention; Korea (ranking ninth globally in mercury emissions) signed off on the convention in 2014 and is preparing for its ratification in 2017 [3].

A fundamental analysis conducted by the Korean Ministry of Environment for the Comprehensive Mercury Management Measures (2016–2020) in preparation for the Minamata convention, showed that the amount of imported mercury in 2011 was around 10.2 t; of this, 1 t was used in dental amalgam [4]. The mercury used in dental amalgam is not being properly disposed of. Instead it is incinerated with other medical waste, indicating the lack of a proper management strategy for the emission and disposal of mercury waste [5]. Korea does not legally mandate mercury separators, and very few have been sold commercially. Based on medical law, extracted teeth are entrusted to a disposal company along with other tissue and incinerated with other medical waste [5].

For Korea to fulfill the regulations of the Minamata convention, the disposal and management processes for mercury-containing waste need to be assessed and controlled. In other words, the disposal process of not only mercury-based thermometers and sphygmomanometers, but also that of dental amalgam, needs to be adequately managed. Specifically, all dental-related amalgam-containing waste, including excess mercury, excess amalgam that was not used in tooth fillings, amalgam that was removed from teeth, and extracted amalgam-containing teeth, must be separated and disposed of adequately.

Some studies in other countries have assessed amalgam-related mercury usage in dental clinics, including the amount of mercury used in amalgam, the level of mercury emission, and the number of mercury fillings in the teeth of the general population [6,7]. However, in Korea, previous studies were mostly restricted to analyses of the mercury concentration in the human body from dental fillings or the removal of amalgam fillings/disposal of amalgam [8,9,10,11,12]. Therefore, studies investigating the amount of mercury used in dental amalgam, its circulation, and its disposal are needed.

Despite the continuous decrease in amalgam usage (due to the risks for human health and the environment related to mercury), the amount of mercury disposed of by dental clinics in Korea is expected to remain stable over the next few years, owed to the long lifespan of amalgam (about 10 years) and the number of extracted amalgam fillings.

In this study, we assessed the amalgam filling rate and amalgam weight of extracted teeth from a convenience sample of dental clinics in Korea. Using data from the National Health Insurance on the number of extracted teeth per year [13], we estimated the levels of mercury emission into the environment in order to provide foundational data for establishing an appropriate management protocol for extracted mercury-containing amalgam fillings.

## 2. Materials and Methods

### 2.1. Study Design and Data

This cross-sectional study was performed at a dental clinic of a general hospital (City of Daegu), a dental hospital (City of Daegu), and five private dental clinics (one in the South and four in the North Gyeongsang Province), which were selected using convenience sampling between November 2014 and September 2015.

As most dental amalgam fillings are performed on posterior teeth (premolars and molars), we collected the teeth extracted at these dental clinics that were claimed for insurance under the code “extraction-posterior teeth (U4413)”. The extracted teeth (*N* = 1208) were visually verified and classified as either anterior (incisors and canines) or posterior teeth by a dental hygienist. Then, the amalgam filling rate of the posterior teeth was assessed. The amalgam-filled posterior teeth were cut out with a dental disk bur, and the amalgam was separated for weight measurement. The removal of amalgam was visually confirmed by color.

As extracted tooth samples are not defined as personally identifiable information, the Yeungnam University’s institutional review board (IRB) determined that the study protocol was exempt from review (No. 7002016-E-2014-001). It is common practice for Korean dental clinics to collect extracted teeth for two months prior to disposal by waste management companies. Because the study samples (used in this study) were extracted teeth that had been kept for disposal, it was impossible to obtain consent from the patients. Accordingly, the IRB of Yeungnam University approved a waiver of informed consent.

### 2.2. Data Analysis

The amalgam filling rate at each dental institution is shown as the arithmetic mean, and the amalgam weight is presented as the geometric mean and 95% confidence interval (CI). We calculated the weight for each type of dental institution separately because the amalgam filling rate differs between them.

Data from the National Health Insurance (the number of extracted posterior teeth per year and the number of dental clinics in Korea) [13], dental institution weights, and the results of our amalgam analyses (amalgam filling rate and mean amalgam weight) were used to calculate the estimated amount of mercury emitted from extracted teeth into the environment, using the following equation: annual number of extracted posterior teeth × amalgam filling rate (%) × mean amalgam weight (g) × 0.5 (the proportion of mercury in amalgam). Dental amalgam contains silver, tin, copper, and approximately 50% mercury by weight [6,14].

According to data from the National Health Insurance, there were 265 general hospitals, 217 dental hospitals, and 16,575 dental clinics in Korea as of November 2015. As our study included a general hospital, a dental hospital, and five dental clinics, the dental institution weights were 265/1, 217/1, and 16,575/5 for the general hospital, dental hospital, and dental clinics, respectively.

All data analyses were performed with SPSS ver. 18.0 (SPSS Inc., Chicago, IL, USA).

## 3. Results

### 3.1. Amalgam Filling Rate and Weight by Dental Institution

Of the total number of extracted posterior teeth during the study period (*N* = 1208), 138 (11.4%) were amalgam-filled. After applying the dental institution weights, the overall amalgam filling rate was 9.9% (Table 1).

The amount of amalgam extracted from the amalgam-filled posterior teeth was 44.0 g, and the geometric mean of the individual amalgam weight was 0.2 g (Table 2).

### 3.2. Estimated Mercury Emission from Extracted Dental Amalgam

We then calculated the estimated mercury emission from the incineration of extracted amalgam-filled posterior teeth, as follows: 4,280,000 posterior teeth × 11.42% × 0.20 g × 50% ≈ 48.86 kg (95% CI 41.53–58.63 kg); after applying the dental institution weights, it was: 4,280,000 posterior teeth × 9.94% × 0.20 g × 50% ≈ 42.53 kg (95% CI 34.11–52.17 kg). Therefore, we estimate that about 42.53–48.86 kg of mercury is incinerated along with extracted teeth without a proper disposal protocol.

## 4. Discussion

The government of Korea has revised the legislation (taking effect from 2020 onwards) related to the stepwise reduction of mercury use and/or the prohibition of the synthesis, export, and import of mercury-containing products (except for certain products for military or research use) in order to lay a foundation for the efficient management of mercury and to prepare for the Minamata convention coming into effect [15]. Moreover, there are ongoing plans to reduce mercury emissions by identifying the emission levels of various sources (e.g., atmosphere or water) to set appropriate emission limits and to establish an eco-friendly management system for mercury-containing products [16]. However, even with the revised legislation and management protocols, the levels of mercury emission and disposal will not decrease in the near future as a large amount of mercury has been used to date. Therefore, a management protocol for the proper collection and disposal of mercury-containing products must be urgently established.

Our results indicate that there is 0.1 g Hg in an average amalgam filling. However, Swedish Chemicals Inspectorate reported 0.6 g Hg in an average amalgam filling in Sweden [6]. One explanation for this difference is that they assumed a medium-sized three-surface amalgam filling in Sweden, whereas we did not limit the size or surface of amalgam filling. Furthermore, bulk dental mercury and bulk amalgam alloy are generally used, unlike the pre-capsulated alloy used in Sweden. As such, we can mix amalgam more closely to the exact amount needed. Although a certain portion of non-contact amalgam (excess mix left over at the end of a dental procedure) waste is collected separately, most dental amalgam is incinerated along with other medical waste [4]; importantly, the amount being incinerated has not yet been reported. The estimated amount of mercury being incinerated with the extracted posterior teeth is about 42.53–48.86 kg per year. Therefore, as is done in the United States, we need to separate extracted teeth based on whether they have amalgam restorations, and such amalgam-containing teeth should not be incinerated [17]. Nevertheless, the incineration of medical waste is responsible for 20%, or 65 t, of mercury released into the air annually [18]. The ADA strongly recommends mercury recycling as a best management practice [19].

The Comprehensive Mercury Management Measures (2016–2020) recommend the gradual reduction of dental amalgam use from 2020 onwards [16]. In fact, dental amalgam usage has been steadily decreasing in Korea due to concerns about human health and the environment. The number of amalgam filling procedures has been steadily decreasing from 4.1 million in 2012 to 3.54 million in 2013 and 3.09 million in 2014 [13].

The annual amount of mercury usage in the form of dental fillings in 15 countries of the European Union (EU) and the European Free Trade Association (EFTA) has been estimated to be about 1300–2200 t [20,21]. In Sweden, the estimated amount of dental filling-based mercury was 40 t in 2004 [6]. However, since a restriction on amalgam usage in dental clinics was implemented in 2003, the amount of mercury used in dental clinics has been reduced to about 100 kg [6], and the use of mercury was forbidden by law in 2009. In France, the estimated amount of mercury in the form of dental fillings is about 100 t or about 2 g per person. Similarly, the estimated amount of mercury in the form of dental fillings in the USA is about 1000 t [6].

To the best of our knowledge, this is the first study to assess the average amount of mercury in dental fillings in the Korean population. Future studies should determine the amount of amalgam in teeth, the levels of mercury emitted from dental amalgam, both in extracted teeth and from the decay of deceased individuals (from both incineration and burial), and the amount of excess mercury and non-contact recyclable amalgam.

The limitations of this study include the convenience sampling method, which was based on a restricted geographical region within Korea, and the cross-sectional study design that only accounted for the size of the medical institution. We did not calculate the sample size of this study. Furthermore, using data from the National Health Insurance may have introduced errors resulting from multiple insurance claims. Last, the amount of mercury in amalgam in this study represents the initial amount and does not consider the amount of lost mercury (e.g., from manipulation or mastication). A nationwide longitudinal study using random sampling and an adequate sample size reflecting the number of dental institutions in Korea should be performed in the future.

## 5. Conclusions

In conclusion, the estimated amount of mercury being incinerated with extracted posterior teeth at Korean dental institutions is about 42.53–48.86 kg/year.

## Figures and Tables

**Table 1:** Amalgam filling rate by dental institution.

Dental Institution	*N*	Extracted Teeth (*N*)	Teeth with Amalgam Fillings (*N*)	Amalgam Filling Rate	Dental Institution Weight
General hospital	1	124	24	19.35	265
Dental hospital	1	94	17	18.09	217
Dental clinic	5	990	97	9.79	16,575
Total	7	1208	138	11.42	3797

**Table 2:** Weight of amalgam by dental institution.

Dental Institution	*N*	Teeth with Amalgam Fillings (*N*)	Weight of Amalgam (g)	Geometric Mean (g)	95% CI
General hospital	1	24	6.86	0.19	0.13–0.28
Dental hospital	1	17	4.46	0.14	0.07–0.25
Dental clinic	5	97	32.67	0.22	0.18–0.27
Total	7	138	43.99	0.20	0.17–0.24

CI, confidence interval.
